# Identification and Characterization of an R-M System in *Paracoccus denitrifican* DYTN-1 to Improve the Plasmid Conjugation Transfer Efficiency

**DOI:** 10.4014/jmb.2402.02041

**Published:** 2024-07-26

**Authors:** Yunpeng Shi, Wenyan Cao, Zhiping Zheng, Sha Xu, Lijuan Chai, Shenghu Zhou, Yu Deng

**Affiliations:** School of Biotechnology and Key Laboratory of Industrial Biotechnology of Ministry of Education, Jiangnan University, Wuxi 214122, P.R. China

**Keywords:** Restriction-modification system, *Paracoccus denitrificans*, DNA methylase, HNH endonuclease, plasmid artificial modification

## Abstract

*Paracoccus denitrificans* has been identified as a representative strain with heterotrophic nitrification-aerobic denitrification capabilities (HN-AD), and demonstrates strong denitrification proficiency. Previously, we isolated the DYTN-1 strain from activated sludge, and it has showcased remarkable nitrogen removal abilities and genetic editability, which positions *P. denitrificans* DYTN-1 as a promising chassis cell for synthetic biology engineering, with versatile pollutant degradation capabilities. However, the strain’s low stability in plasmid conjugation transfer efficiency (PCTE) hampers gene editing efficacy, and is attributed to its restriction modification system (R-M system). To overcome this limitation, we characterized the R-M system in *P. denitrificans* DYTN-1 and identified a DNA endonuclease and 13 DNA methylases, with the DNA endonuclease identified as HNH endonuclease. Subsequently, we developed a plasmid artificial modification approach to enhance conjugation transfer efficiency, which resulted in a remarkable 44-fold improvement in single colony production. This was accompanied by an increase in the frequency of positive colonies from 33.3% to 100%. Simultaneously, we cloned, expressed, and characterized the speculative HNH endonuclease capable of degrading unmethylated DNA at 30°C without specific cutting site preference. Notably, the impact of DNA methylase M9 modification on the plasmid was discovered, significantly impeding the cutting efficiency of the HNH endonuclease. This revelation unveils a novel R-M system in *P. denitrificans* and sheds light on protective mechanisms employed against exogenous DNA invasion. These findings pave the way for future engineering endeavors aimed at enhancing the DNA editability of *P. denitrificans*.

## Introduction

*Paracoccus denitrificans*, a heterotrophic nitrification-aerobic denitrification bacteria (NH-AD), was initially identified by Robertson *et al*. in the late 20th century [1]. These strains exhibit the unique capability to utilize O_2_ and NO_3_^-^ in the environment under aerobic conditions, or intermediate metabolites generated during denitrification as electron acceptors for denitrification reactions. The outcome of these reactions is the production of gaseous nitrogen, coupled with the simultaneous removal of chemical oxygen demand (COD) from wastewater [2]. We have isolated *P. denitrificans* DYTN-1 strain from activated sludge and sequenced its entire genome, revealing the strain’s remarkable ability to reduce total nitrogen content in wastewater from 100 mg/l to below 8.4 mg/l within 72 h, and showcasing its exceptionally high ammonia nitrogen degradation efficiency [3]. Consequently, *P. denitrificans* DYTN-1 has emerged as a promising chassis cell for synthetic biology engineering, one that provides versatile degradation capabilities for diverse pollutants and could serve as a microbial cell factory for chemical production under anaerobic conditions.

Currently, conjugation stands as the most efficient gene editing method for *P. denitrificans*, enabling specific site editing of the strain and precise regulation of target gene expression on plasmids [4]. However, early findings indicated a barrier of restriction enzymes in *Paracoccus* that have so far hindered efficient conjugation. To enhance the modification efficiency of *P. denitrificans*, de Vries *et al*. designed a high-throughput screening procedure to obtain mutant strains more efficiently [5]. Triparental mating experiments with receptor strain PD1222 (*Pde*I^-^) revealed a fourfold increase in conjugation efficiency after mutagenesis [5]. Compared to the wild-type strain, PD1222 lost its digestion resistance to BglI, BamHI, Sau3A, and MboI [5]. Moreover, the control experiments with methylation-sensitive restriction enzymes indicated the presence of a modification system with an action site (nGATCn) in *Paracoccus* [5]. All this evidence suggests the existence of an R-M system in *P. denitrificans*. Although advancements have been made in previous studies, the key R-M enzymes remained unclear, limiting the accurate application of genetic engineering methods to further enhance conjugation transfer efficiency.

The prevalent microbial self-protection system, or R-M system, is found in various microorganisms. This system specifically cleaves phage nucleic acid sequences to counteract the invasion of foreign genetic material [6]. The R-M system comprises endonucleases and cognate DNA methylases responsible for the functional cutting and methylation modification of recognized exogenous DNA, respectively [7, 8]. Exogenous DNA lacking methylation modifications by cognate DNA methylase can be specifically cleaved by endonucleases. In contrast, endogenous methylated DNA is resistant to cleavage [9-11]. Until now, R-M systems have been widely discovered in the related species of *P. denitrificans*. For example, type II R-M systems have been extensively described for *Rhodopseudomonas sphaeroides* [12-18]. In this R-M system, RsrI and RshI with recognition sequences (G|AATTC) and (CGAT|CG) were identified as isoschizomers of EcoRI and PvuI, respectively. Other endonucleases like RsaI and RsrlI were also identified from the genome with the recognition sequences of (GT|AC) and (CG|G(A/T)CCG) [4]. Furthermore, M.RsrI was identified as the methyltransferase for N-6-methylation of adenine, leading to protection against these restriction enzymes. Thus, it is essential and feasible for identifying the native R-M system genes from *P. denitrificans*.

In summary, the undisclosed R-M system of *P. denitrificans* DYTN-1 hinders synthetic biology engineering in this bacterium. In this study, we identified the enzymes of the R-M system in *P. denitrificans* DYTN-1. Utilizing the identified enzymes, we devised a Plasmid Artificial Modification (PAM) approach to boost plasmid conjugation transfer efficiency by methylation modification before DNA enters the receptor cell. Additionally, we purified and characterized the identified HNH endonuclease, offering valuable insights into the restriction barrier [19] of *P. denitrificans* and laying the foundation for the development of synthetic biology in these strains.

## Materials and Methods

### Strains, Medium, and Culture Conditions

*E. coli* S17-1 λ pir was employed for plasmids construction and served as the host for the PAM process, while *E. coli* ER2566 was utilized to express and purify the HNH endonuclease. Luria Bertani (LB) medium was used for both plasmid construction and PAM. The HNH endonuclease was expressed at 20°C with continuous shaking at 220 rpm using Terrific Broth (TB) medium (24 g/l yeast extract, 12 g/l tryptone, 0.4% glycerol, 12.47 g/l K_2_HPO_4_ and 2.31 g/l KH_2_PO_4_) in 250 ml triangular flasks. The media were supplemented with 100 μg/ml spectinomycin, 100 μg/ml ampicillin, 34 μg/ml chloramphenicol, and 50 μg/ml kanamycin when necessary. Details of the strains and plasmids used in this study are provided in Table 1.

### Plasmid Construction

**Fig. 1:**
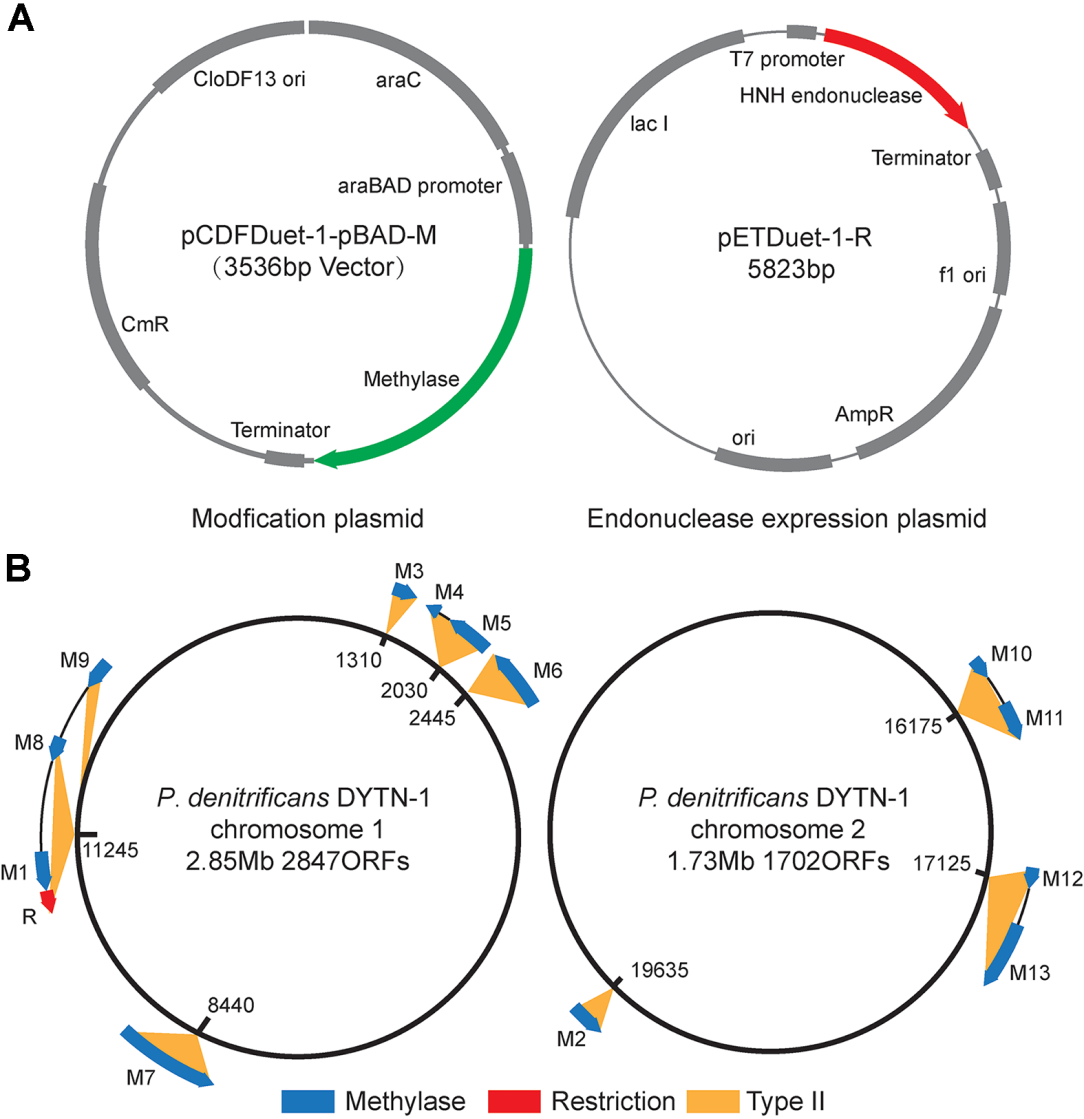
Depicts the architecture of the clonal plasmid and maps the distribution of the R-M system within the genome of *P. denitrificans* DYTN-1. (**A**) Plasmids designed for the expression of R-M system-related genes. pCDFDuet- 1-pBAD-M illustrates the structure of all 13 methylase expression plasmids, while pETDuet-1-R depicts the structure of the HNH endonuclease expression plasmid. (**B**) The predicted distribution of R-M system-related genes on the *P. denitrificans* DYTN-1 genome according to REBASE.

Restriction enzymes and T4 DNA ligase were purchased from Takara (China). ClonExpress Ultra One Step Cloning Kit used for plasmid construction was purchased from Vazyme Biotech (China). All 13 DNA methylase genes were amplified from *P. denitrificans* DYTN-1 by corresponding primer pairs of Mn-U/Mn-D. The amplified DNA methylase genes were cloned into the linearized plasmid pCDFDuet-1-pBAD, generating plasmid series pCDFDuet-1-pBAD-M (M represents different methylase genes M1-M13) (Fig. 1A). The plasmid was equipped with an arabinose inducer promoter pBAD, which can control the expression of DNA methylases under arabinose induction. The plasmids pCDFDuet-1-pBAD-M were transformed into *E. coli* S17-1 λ pir for amplification and further PAM. To collect enzyme, the HNH endonuclease genes were amplified from *P. denitrificans* DYTN-1 by primer pairs of R-U/R-D and cloned into NcoI/BamHI sites of pETDuet-1, generating plasmid pETDUet-1-R (Fig. 1A). pETDUet-1-R was constructed in *E. coli* ER2566 cells that were induced to express DNA methylase to inhibit the destruction of endonuclease on the genome. Primers used in this study are listed in Table 2.

### PAM of the Shuttle Plasmid and Conjugation Transfer

The shuttle plasmid was transformed into *E. coli* S17-1 λ pir strain containing pCDFDuet-1-pBAD-M. The positive clones were then cultured at 37°C, 250 rpm until the OD_600_ hit 0.6-0.8. A final concentration of 10 mM arabinose was then added to induce DNA methylase expression. After 16 h of cultivation, the shuttle plasmids underwent methylation modification by the expressed DNA methylases. *P. denitrificans* DYTN-1 was preliminarily cultivated at 30°C and 220 rpm. The culture was then centrifuged at 5,000 ×*g* for 3 min and washed twice with antibiotic-free LB medium. *E. coli* S17-1 λ pir and *P. denitrificans* DYTN-1 were mixed at a 3:10 ratio of cell mass in the same tube, and statically incubated for 1 h at 37°C. Subsequently, the tube was shaken vigorously to disrupt the sexual pili connection between the two bacteria, stopping gene transfer. Finally, 0.1 ml of conjugated bacterial solution was coated on an agar plate containing spectinomycin and kanamycin to screen positive clones, and the number of single colonies on the plate and the proportion of positive single colonies were determined.

### Expression and Purification of HNH Endonuclease

The pCDFDuet-1-pBAD-M9 plasmid was transformed into *E. coli* ER2566 [20] to express DNA methylase. Then, pETDuet-1-R was introduced into the same *E. coli* ER2566 strain, obtaining strain *E. coli* ER2566 M9+R. This strain can express both DNA methylase M9 and 6xHis-tagged HNH endonuclease under arabinose and IPTG induction, respectively. *E. coli* ER2566 M9+R was inoculated into liquid TB medium containing ampicillin and chloramphenicol. A final concentration of 10 mM arabinose was added and the cells were shaken at 250 rpm and 37°C until the OD_600_ reached 0.6. Then, a final concentration of 0.6 mM IPTG was added, and the cells were shaken at 200 rpm and 20°C for 48 h. After induction, cells were collected by centrifuging at 8,000 ×*g* for 10 min.

The collected cells were fully resuspended in a buffer solution (10 mM Tris HCl, pH 7.4, 250 mM NaCl, 5%glycerol, 0.15% Triton X-100, 5 mM imidazole, 1 mM benzylsulfonyl fluoride). Then, the cells were lysed using a homogenizer, and the insoluble components were removed by centrifugation at 10,000 ×*g* for 30 min. Following that, the collected supernatant was filtered using a 45 mm aqueous phase filter membrane. After filtration, Ni-affinity chromatography was performed using the AKTA Avant system (Cytiva) to obtain the preliminary purified endonuclease. Three buffer solutions were employed in the Ni-affinity chromatography process: an equilibrium buffer (10 mM Tris HCl [pH = 7.4], 250 mM NaCl, 5% glycerol, 0.15% Triton X-100, 5 mM imidazole), an elution buffer (10 mM Tris HCl [pH = 7.4], 250 mM NaCl, 5% glycerol, 0.15% Triton X-100, 400 mM imidazole), and a rinse buffer (10 mM Tris HCl [pH = 7.4], 250 mM NaCl, 5% glycerol, 0.15% Triton X-100, 20 mM imidazole). Following the consolidation of elution peaks containing HNH endonuclease, a 10 kDa ultrafiltration tube was used for dialysis and storage buffer (10 mM Tris HCl [pH = 7.4], 250 mM NaCl, 50% glycerol, 0.15% Triton X-100, 0.1 mM EDTA, 1 mM DTT). The entire purification operation must be completed on ice, or at 4°C. Bradford colorimetry was used to determine the protein concentration. Then, molecular sieve chromatography was performed using the AKTA Avant system on the preliminarily purified HNH endonuclease. We used a collection tube to collect the washed-off protein separately and concentrate them with ultrafiltration tubes again. The target protein was determined by SDS-PAGE and then stored at -20°C for further use.

### Characterization of the HNH Endonuclease

The unmethylated PIND4-abrB plasmid was used to test the digestion performance of the purified HNH endonuclease at gradient temperature. In doing so, 1 μg plasmid and 1 μg enzyme were added in 20 μl buffer K (10 mM Tris-HCI [pH 7.5], 10 mM MgCl_2_ 1 mM dithiothreitol, and 100 mM KCl) for cutting for 24 h. Then, the digested plasmid DNA was subjected to agarose gel electrophoresis. The optimal digestion temperature range of the HNH endonuclease could be inferred based on the gel result. At the optimum temperature, the HNH endonuclease digestion performance was tested over 2 h.

## Results

### Screening of Natural R-M System from *P. denitrificans* DYTN-1

*P. denitrificans* DYTN-1, being a natural strain isolated from activated sludge without available genomic sequencing and gene annotation information, prompted the completion of genomic sequencing as the initial step. The genome comprises 2 chromosomes and 1 plasmid, with chromosome 1, chromosome 2, and the plasmid measuring 2,853,092 bp, 1,730,123 bp, and 653,817 bp, respectively. In total, the genome contains 5,227 genes.

The R-M system, a common feature in prokaryotes, serves the primary function of resisting and eliminating foreign DNA, such as that of bacteriophages or plasmids [21], representing a fundamental form of natural immune defense [22]. To identify potential endonucleases and methylases in *P. denitrificans* DYTN-1, the genomic sequencing information was uploaded to the NCBI (NCBI ID: CP096593), and then inputted into REBASE (http://REBASE.neb.com/REBASE/REBASE.html) [23]. REBASE identified 13 potential methylases and a potential endonuclease from the *P. denitrificans* DYTN-1 genome (Fig. 1B, Table 3, and Supplementary Table 1). The built-in algorithm of REBASE indicated that the DNA endonuclease identified is an HNH endonuclease, which belongs to the type II restriction-modification system.

### Enhancing the Conjugation Transfer Efficiency of *P. denitrificans* DYTN-1 by PAM

**Fig. 2:**
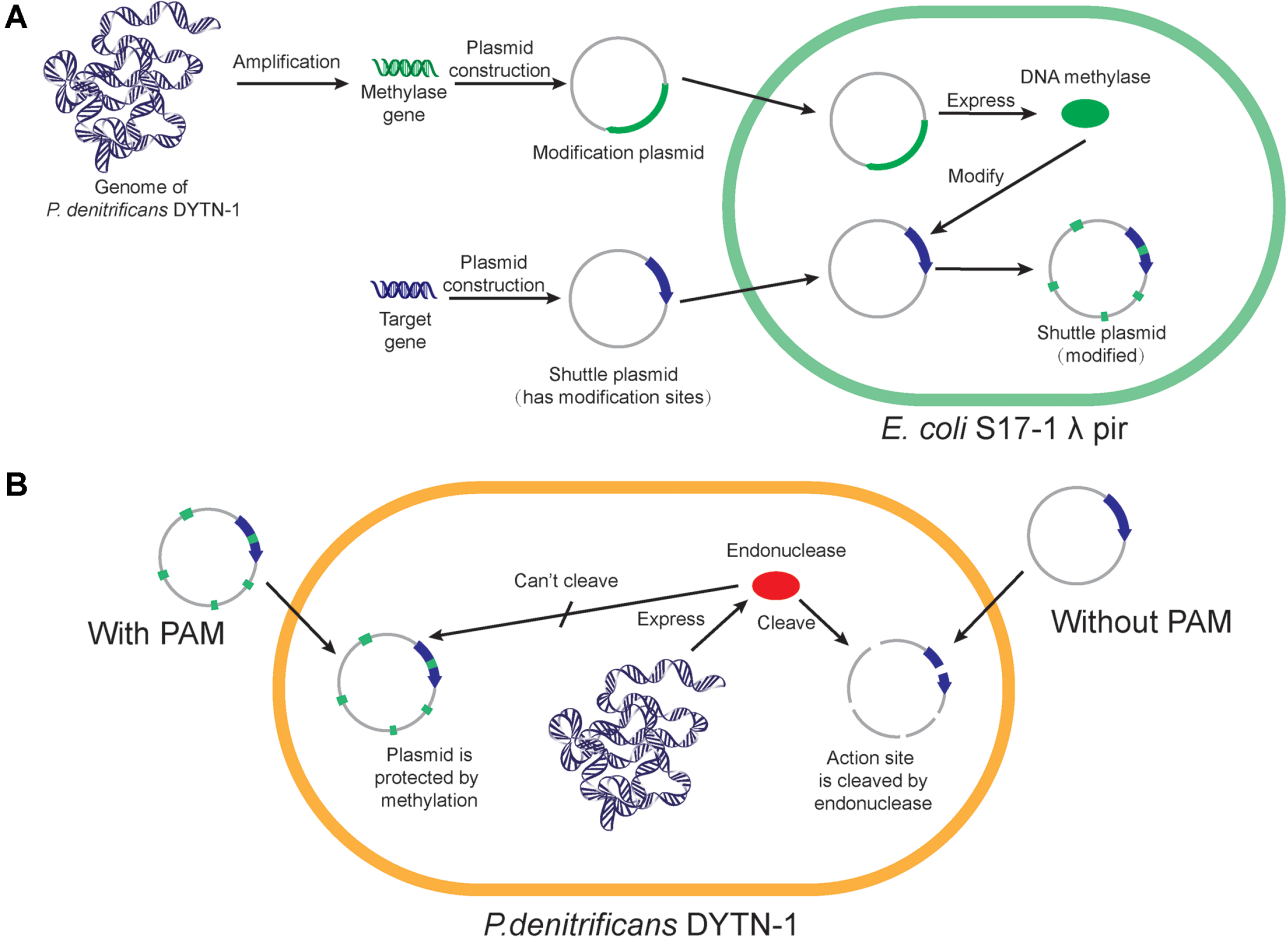
The PAM execution process. (**A**) Modification of the shuttle plasmid involved in amplification of methylase genes from *P. denitrificans* DYTN-1. These genes were used to construct modification plasmids, which were then transformed into *E. coli* S17-1 λ pir for methylase expression. Subsequently, the shuttle plasmid was introduced, and methylated by the overexpressed methylases. (**B**) Protection mechanism of PAM for the shuttle plasmid: After PAM, methylation protected the endonuclease cleavage site, preventing cleavage of the shuttle plasmid. A shuttle plasmid lacking PAM is susceptible to endonuclease cleavage, resulting in low conjugation efficiency.

Restrictive endonucleases recognize and cleave specific sequences of 4-8 bp. However, the recognition ability of these enzymes will not work when these sequences are modified by methyltransferase [24]. Hence, expressing DNA methyltransferases in a donor strain can methylate the shuttle plasmid during replication within the same strain. This methylated shuttle plasmid is protected from cleavage during conjugation and replication in the recipient bacterium, potentially leading to a significant improvement in conjugation transfer efficiency. This approach is referred to as PAM (Fig. 2) [25]. The start codon of all R-M system genes was optimized to ATG to facilitate expression in *E. coli*. During PAM, constitutive expression of heterogeneous methylases could adversely impact host growth, especially in the early stages. Therefore, the pCDFduet-1-pBAD plasmid, with an arabinose promoter featuring extremely low background expression, was chosen. This allows for the induction of heterogeneous methylase expression in *E. coli* S17-1 λ pir after reaching a sufficient growth stage, reducing the pressure caused by methylation on the host.

**Fig. 3:**
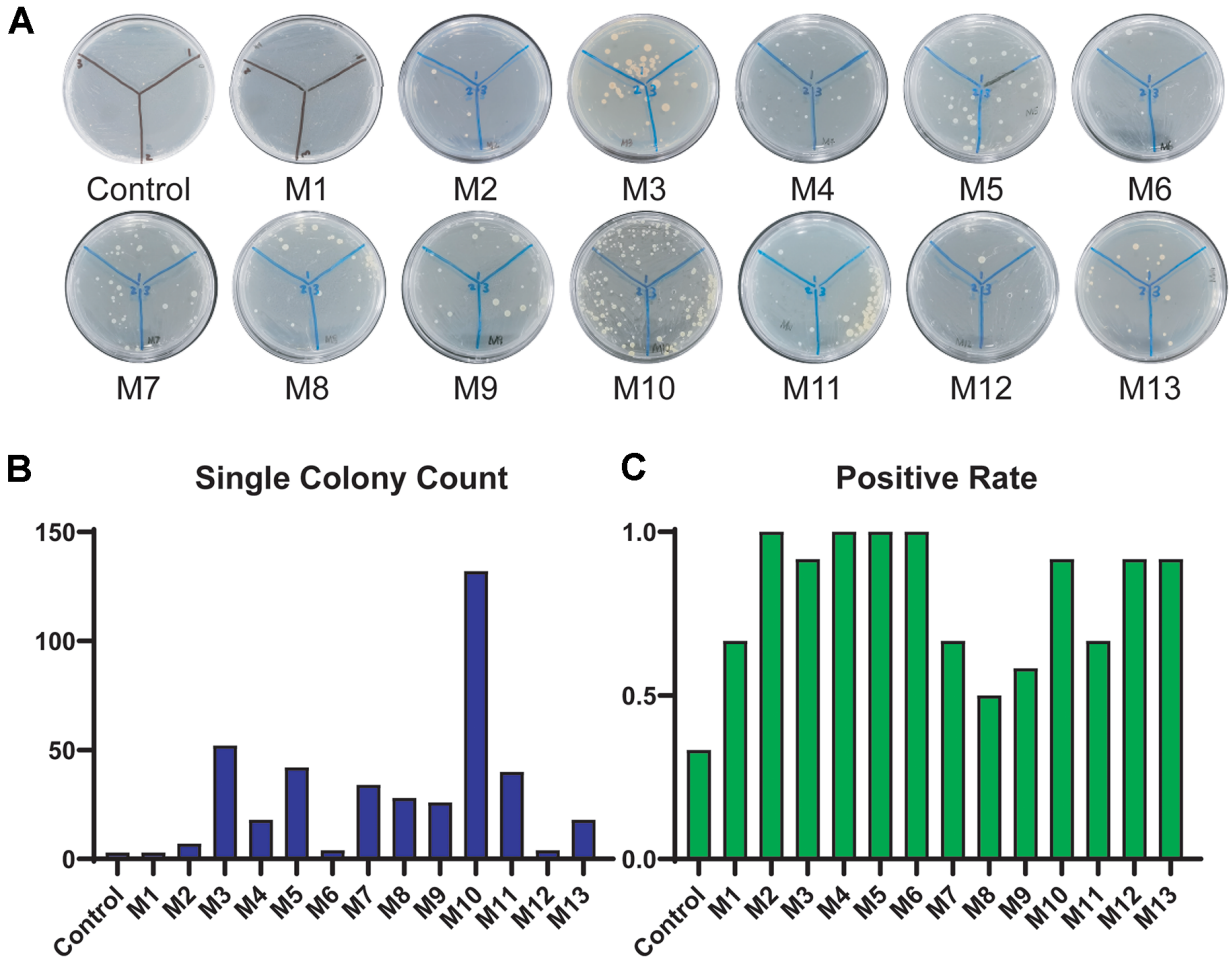
The influence of PAM on conjugation. (**A**) The formation of single colonies during conjugation was observed after 24 h of modification with different methylases (the Control group lacked modification plasmid in the donor strain). (**B**) Represents the count of single colonies, serving as a measure of conjugation efficiency. (**C**) Indicates the positive rate of the resulting single colonies. Twenty-four single colonies on each plate were selected for colony PCR and the plasmid was sequenced to determine the positive rate of the strains. The percentage of positive bacteria verified by colony PCR to the total number of verified colonies represents the positive rate.

Obtaining single colonies by directly conjugating the shuttle plasmid PIND4-abrB into *P. denitrificans* DYTN-1 proved challenging (Fig. 3A). However, following arabinose-induced expression of DNA methylases, a significant improvement in conjugation transfer efficiency was observed compared to conjugation without PAM. All DNA methylases contributed to enhancing conjugation transfer efficiency, as evidenced by a substantial increase in single colony count (from 3 to 132, a 44-fold enhancement) (Fig. 3B) and positive rate (from 33.3% to 100%)(Fig. 3C). Among these methylases, M2, M3, M4, M5, M6, M10, M12, and M13 increased the positivity rate to over 90%. Other methylases also elevated the positive rate to approximately 50% to 67%, suggesting that completing PAM on the plasmid before conjugation enhances the overall conjugation efficiency.

The modification activity of methylases is coupled with the cleavage function of DNA endonucleases, indicating that the efficacy of methylase modification is contingent upon the abundance of the corresponding DNA endonuclease cleavage sites on the plasmid. A higher density of recognized sites on a given exogenous plasmid correlates with a more pronounced modification effect. Through an analysis of the methylation sites on the plasmid PIND4-abrB, 21 putative modification sites for methylase M9 (GANTC) and 10 sites for M11 and M13 (C/TGGCCG/A) were identified. The conjugation results demonstrated that methylation by M11 and M13 yielded higher conjugation transfer efficiency compared to M9 (Figs. 3B and 3C). Thus, we posit that the variability in methylase modification effects may be attributable to the plasmid sequences and yet-to-be-discovered DNA endonucleases. Furthermore, certain DNA endonucleases may not have high recognition specificity, so each of the DNA methylases can have a certain effect on improving transfer efficiency.

### Expression and Purification of HNH Endonuclease

Initially, *E. coli* BL21 (DE3) and JM109 were chosen as expression hosts to produce HNH endonuclease, but transformants could not be obtained. This issue was likely attributed to the expression of DNA endonucleases, leading to cleavage of the host genome DNA and the subsequent demise of the host cell. This challenge in producing and characterizing DNA endonucleases is well-documented [26]. To overcome this limitation, the *E. coli* mutant strain ER2566 (mcrC mrr) was selected as host strain since it can tolerate DNA damage caused by non-specific nucleases.

*E. coli* ER2566 is a strain specifically designed to express and purify DNA-active enzymes, such as endonucleases and methyltransferases. By introducing genes from *E. coli* K-12, the cellular response to DNA damages, like the effect of non-specific nucleases, modification-dependent enzymes, and the T1 phage, were altered. Additionally, the λDE3 prophage is absent, and T7 RNA polymerase has been integrated into *lacZ*, thereby preventing cell lysis induced by the expressed DNA endonucleases [20]. Therefore, *E. coli* ER2566 is suitable for expressing and purifying DNA endonucleases.

**Fig. 4:**
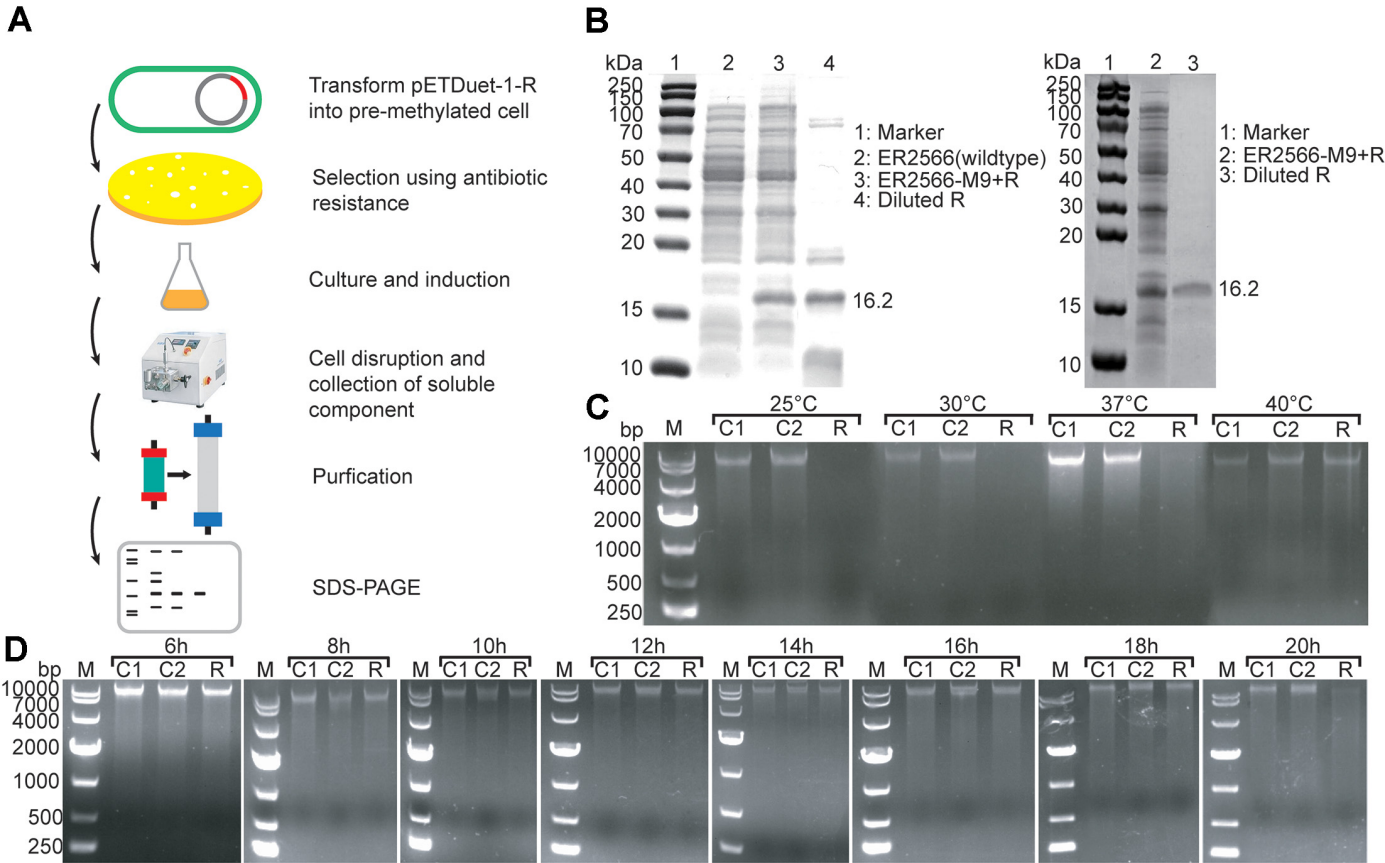
Purification of the HNH endonuclease and its cleavage effect on plasmid PIND4-abrB. (**A**) Expression and purification procedure of HNH endonuclease. (**B**) SDS-PAGE of HNH endonuclease R after Ni-affinity chromatography (left) and after molecular sieve chromatography (right). A visible 16.2 kDa target protein band present on the gel (indicated by arrow). (**C**) Unmethylated plasmid PIND4-abrB (9,933 bp) cleaved by purified HNH endonuclease for 24 h under a gradient temperature. (**D**) PIND4-abrB was cleaved by the HNH endonuclease at 30°C for varying durations. M: DNA marker; C1: Control 1 without the addition of endonuclease, only plasmid in buffer K; C2: Control 2 with the endonuclease inactivated by heating to 95°C and incubating for 15 min before addition.

However, direct expression of DNA endonucleases using the T7 promoter in *E. coli* ER2566 could not yield the correct transformant, indicating that the leakage expression of endonuclease can kill host cells not protected by preliminary methylated modification [27-29]. To address this issue, pCDFDuet-1-pBAD-M plasmids were transferred into *E. coli* ER2566, and the expression of DNA methylases was induced to methylate modification and prepare competent cells. Subsequently, the pETDuet-1-R plasmid was transformed into *E. coli* ER2566 (pCDFDuet-1-pBAD-M) to express HNH endonuclease using IPTG induction (Fig. 4A). Finally, the best match between HNH endonuclease and methylases was determined, obtaining the optimum expression strain, named *E. coli* ER2566-M9+R. Except for M9, the transfer of plasmid pETDuet-1-R into strains expressing other methylases does not yield single colonies with correct sequencing. These findings suggest the probable presence of additional DNA endonucleases in *P. denitrificans* DYTN-1, which are likely to be associated with other DNA methylases rather than M9. The results obtained for methylase M9 and HNH endonuclease R are consistent with the general distribution principle of R-M systems, where DNA endonucleases and methylases are often found on the same chromosome and are relatively close in distance.

The HNH endonuclease was separated and purified using a Ni-NTA affinity chromatography column. The majority of impurity proteins were eliminated through Ni-affinity chromatography, yet evident heterologous proteins remained in the range of 70-100 kDa and <15 kDa. Subsequently, molecular sieves were employed for further purification of the HNH endonuclease, resulting in a substantial enhancement of enzyme purity (Fig. 4B). The ultimate concentration of purified HNH endonuclease reached 2.3 mg/ml, with a molecular weight of 16.2 kDa.

### Characterization of HNH Endonuclease

To detect the activity of the HNH endonuclease, the purified enzyme was used to cleave the PIND4-abrB plasmid. The selection of PIND4-abrB for characterizing the HNH endonuclease was based on its notable enhancement in DNA conjugation efficiency following the PAM process, indicating the plasmid's sensitivity to HNH endonuclease (Fig. 2). The HNH endonuclease exhibited a digestion activity under 20~37°C, but at temperatures higher than 40°C, the activity is lost (Fig. 4C). Subsequently, the outcomes of HNH endonuclease cleavage at 30°C for various durations demonstrated complete digestion of the plasmid by the HNH endonuclease after 20 h in buffer K (Fig. 4D). This result suggests that, under the given conditions, the identified HNH endonuclease does not exhibit strict cleavage site specificity.

In the plasmid conjugation transfer process, the positive single colonies need about 48 h to fully grow. In this scenario, if the plasmid DNA is not kept intact during this 48 h period, it will greatly interfere with the entering efficiency of exogenous DNA in *P. denitrificans* DYTN-1. Thus, our result strongly supports the inference that the endogenous R-M system of *P. denitrificans* DYTN-1 is a key factor hindering efficient transfer and stability of plasmids. Simultaneously, the low selectivity of HNH endonuclease also explains the reason why different DNA methylation enzymes observed in previous experiments have a significant enhancing effect on the conjugation efficiency of *P. denitrificans* DYTN-1.

## Discussion

The stable expression of foreign genes in new hosts highly depends on the presence of R-M systems. Since DNA methylation takes a certain amount of time, R-M systems can quickly degrade foreign genes before they can be stably inherited after specific DNA loci have been methylated. In our previous study, we found that the plasmid conjugation efficiency is low and very unstable when different plasmids are transferred into *P. denitrificans* DYTN-1, indicating the existence of a high restriction barrier. Thus, to develop *P. denitrificans* DYTN-1 into an efficient chassis cell, it is necessary to overcome the effects of this host-specific R-M system. However, research on using synthetic biology methods to enhance the performance ability of *P. denitrificans* is still in its early stages, and most of the related research focuses on deactivating the R-M system [5]. Therefore, we circumvented this barrier through a PAM method.

The PAM method was first proposed in the 1950s by Arber *et al*. [30]. Following this, Elhai *et al*. discovered three methyltransferases that can jointly enhance the conjugation efficiency of *Anabaena* spp [31]. Furthermore, Suzuki *et al*. systematized the PAM process and applied it to three strains of *Bifidobacterium* and one strain of *Lactococcus*, resulting in a 7-10^5^ fold increase in electroporation efficiency [25]. Therefore, applying PAM to enhance the conjugation efficiency of plasmids in *P. denitrificans* DYTN-1 appeared to be a viable approach. In this regard, we designed a PAM strategy to enhance plasmid conjugation transfer efficiency by completing the methylation of the shuttle plasmid before the start of conjugation, greatly increasing the transfer efficiency. Compared to traditional methods that create mutant strains using chemical mutagenesis to inactivate R-M related genes [32] or selecting plasmid mutants that have lost recognition sites [33], constructing modification strains for PAM has multiple advantages, such as low workload, stable effect, and wide applicability. Furthermore, after replacing the methylase, and using *E. coli* as the methylated modification strain, this method is applicable to strains with R-M systems [25].

The HNH endonuclease characterization results clarified the correspondence between it and methylase. Due to the characteristics of the type II R-M system, we can infer the cleavage site of R based on the modification site of M9. These results provide strong evidence for the reliability of de Vries *et al*.'s hypothesis on the modification system of *P. denitrificans* with (nGATCn) modification characteristics [5]. Meanwhile, the exploration of HNH endonuclease’s cleavage also provides a reference for further exploration of R-M systems in the future (Fig. 4C and 4D). When the receptor cell’s restriction endonuclease cutting sites are known, the more recognition sites on the shuttle plasmid, the lower the bacterial conjugation efficiency [31, 34, 35]. Therefore, evading the restriction endonuclease cutting sites in the shuttle plasmid is a very effective way to improve the conjugation transfer efficiency. Overall, this study identified and overcomes the interference of the endogenous R-M system of *P. denitrificans* DYTN-1 on plasmid transfer, providing the possibility of more complicated synthetic biology strategies, such as the establishment of large plasmid libraries to express different genes or gene clusters with diverse expression levels, thereby expanding the application areas of *P. denitrificans* DYTN-1.

## Supplemental Materials

Supplementary data for this paper are available on-line only at http://jmb.or.kr.



## Figures and Tables

**Table 1:** Strains and plasmids used in this study.

	Descriptions	Sources
Plasmids		
PIND4	ColE1ori,oriT,repA,KamR	[36]
PIND4-abrB	PIND4 backbone, P_1851_ regulate the expression of hao and abrB	This study
pETDuet-1-R	pETDuet backbone, carrying M0K93_11240	This study
pCDFDuet-1-pBAD	CloDF13 ori,araC,CmR	This study
pCDFDuet-1-pBAD-M1	pCDFDuet-1-pBAD backbone, carrying M0K93_11245	This study
pCDFDuet-1-pBAD-M2	pCDFDuet-1-pBAD backbone, carrying M0K93_19635	This study
pCDFDuet-1-pBAD-M3	pCDFDuet-1-pBAD backbone, carrying M0K93_01310	This study
pCDFDuet-1-pBAD-M4	pCDFDuet-1-pBAD backbone, carrying M0K93_02030	This study
pCDFDuet-1-pBAD-M5	pCDFDuet-1-pBAD backbone, carrying M0K93_02035	This study
pCDFDuet-1-pBAD-M6	pCDFDuet-1-pBAD backbone, carrying M0K93_02445	This study
pCDFDuet-1-pBAD-M7	pCDFDuet-1-pBAD backbone, carrying M0K93_08440	This study
pCDFDuet-1-pBAD-M8	pCDFDuet-1-pBAD backbone, carrying M0K93_11290	This study
pCDFDuet-1-pBAD-M9	pCDFDuet-1-pBAD backbone, carrying M0K93_11445	This study
pCDFDuet-1-pBAD-M10	pCDFDuet-1-pBAD backbone, carrying M0K93_16175	This study
pCDFDuet-1-pBAD-M11	pCDFDuet-1-pBAD backbone, carrying M0K93_16260	This study
pCDFDuet-1-pBAD-M12	pCDFDuet-1-pBAD backbone, carrying M0K93_17125	This study
pCDFDuet-1-pBAD-M13	pCDFDuet-1-pBAD backbone, carrying M0K93_17200	This study
Strains		
*E. coli* S17-1λpir M1+abrB	*E. coli* S17-1λpir carrying plasmids of pCDFDuet-1-pBAD-M1 and PIND4-abrB	This study
*E. coli* S17-1λpir M2+abrB	*E. coli* S17-1λpir carrying plasmids of pCDFDuet-1-pBAD-M2 and PIND4-abrB	This study
*E. coli* S17-1λpir M3+abrB	*E. coli* S17-1λpir carrying plasmids of pCDFDuet-1-pBAD-M3 and PIND4-abrB	This study
*E. coli* S17-1λpir M4+abrB	*E. coli* S17-1λpir carrying plasmids of pCDFDuet-1-pBAD-M4 and PIND4-abrB	This study
*E. coli* S17-1λpir M5+abrB	*E. coli* S17-1λpir carrying plasmids of pCDFDuet-1-pBAD-M5 and PIND4-abrB	This study
*E. coli* S17-1λpir M6+abrB	*E. coli* S17-1λpir carrying plasmids of pCDFDuet-1-pBAD-M6 and PIND4-abrB	This study
*E. coli* S17-1λpir M7+abrB	*E. coli* S17-1λpir carrying plasmids of pCDFDuet-1-pBAD-M7 and PIND4-abrB	This study
*E. coli* S17-1λpir M8+abrB	*E. coli* S17-1λpir carrying plasmids of pCDFDuet-1-pBAD-M8 and PIND4-abrB	This study
*E. coli* S17-1λpir M9+abrB	*E. coli* S17-1λpir carrying plasmids of pCDFDuet-1-pBAD-M9 and PIND4-abrB	This study
*E. coli* S17-1λpir M10+abrB	*E. coli* S17-1λpir carrying plasmids of pCDFDuet-1-pBAD-M10 and PIND4-abrB	This study
*E. coli* S17-1λpir M11+abrB	*E. coli* S17-1λpir carrying plasmids of pCDFDuet-1-pBAD-M11 and PIND4-abrB	This study
*E. coli* S17-1λpir M12+abrB	*E. coli* S17-1λpir carrying plasmids of pCDFDuet-1-pBAD-M12 and PIND4-abrB	This study
*E. coli* S17-1λpir M13+abrB	*E. coli* S17-1λpir carrying plasmids of pCDFDuet-1-pBAD-M13 and PIND4-abrB	This study
*E. coli* ER2566 M9+R	*E. coli* ER2566 carrying plasmids of pCDFDuet-1-pBAD-M9 and pETDuet-1-R	This study

**Table 2:** The primers used in this study.

Primers	Sequences (5' to 3')^a^
M1-U	AACCTTGCCTTCCACTTTCGGCA
M1-D	TGGTGATGGCTGCTGCCTCAAGAGCCCAAAGCTGAAAGG
M1-CU	TGAGGCAGCAGCCATCACCATC
M1-CD	CGAAAGTGGAAGGCAAGGTTTTTATAACCTCCTTAGAGCTCG
M2-U	AACCATGTCCCCGAAACAAGCCTT
M2-D	GCCCTAAGCATTGGGGTCATAGACGG
M2-CU	ATGACCCCAATGCTTAGGGCAGCAGCCATCACCATC
M2-CD	CTTGTTTCGGGGACATGGTTTTTATAACCTCCTTAGAGCTCG
M3-U	AAAACCATGCAGGCCTTCGGCACC
M3-D	ATGGTGATGGCTGCTGCCCTATGCCGCCGCCTTCCG
M3-CU	AGGGCAGCAGCCATCACCATC
M3-CD	CCGAAGGCCTGCATGGTTTTTATAACCTCCTTAGAGCTCG
M4-U	AACCATGACCGTGCATCGCCCC
M4-D	ATGGCTGCTGCCTCAAAGCAGCCGATCTGGC
M4-CU	TGCTTTGAGGCAGCAGCCATCACCATC
M4-CD	GGCGATGCACGGTCATGGTTTTTATAACCTCCTTAGAGCTCG
M5-U	AAACCATGGTGAACCTCAGCTTTCTCTCG
M5-D	ATGGCTGCTGCCTCATTTCAGCATCTCCACCATACT
M5-CU	TGAAATGAGGCAGCAGCCATCACCATC
M5-CD	GCTGAGGTTCACCATGGTTTTTATAACCTCCTTAGAGCTCG
M6-U	AACCATGGGCCAGCATCCTTCC
M6-D	CGTCAGTTGTTCGATGGTTTTCA
M6-CU	AAACCATCGAACAACTGACGGGCAGCAGCCATCACCATC
M6-CD	AAGGATGCTGGCCCATGGTTTTTATAACCTCCTTAGAGCTCG
M7-U	AAACCATGGCCCGCAAACCAATT
M7-D	TGATGGTGATGGCTGCTGCCTCAGGCTGCAAGGCGACC
M7-CU	GGCAGCAGCCATCACCATC
M7-CD	TGGTTTGCGGGCCATGGTTTTTATAACCTCCTTAGAGCTCG
M8-U	AACCGTGACTGATCGCCAGCTGACC
M8-D	CGACATGAGACGGGATTGTTTC
M8-CU	AACAATCCCGTCTCATGTCGGGCAGCAGCCATCACCATC
M8-CD	GCTGGCGATCAGTCACGGTTTTTATAACCTCCTTAGAGCTCG
M9-U	CCATGACGAAGCTTGACATGAAATCC
M9-D	TGATGGTGATGGCTGCTGCCGAAACCGGGAAATCAGTGGG
M9-CU	GGCAGCAGCCATCACCATC
M9-CD	GGTTTTTATAACCTCCTTAGAGCTCG
M10-U	AAACCATGCAGGCTTTCGGCAGC
M10-D	GTGATGGCTGCTGCCTTATGCCGCCGCCTTCCG
M10-CU	CATAAGGCAGCAGCCATCACCATC
M10-CD	GCCGAAAGCCTGCATGGTTTTTATAACCTCCTTAGAGCTCG
M12-U	AACCATGACCGCACAGGAAAATACCC
M12-D	CGCCTTCGGCGGGGGCTGACCTATGCCGCTGCC
M12-CU	GCAGCAGCCATCACCATCAT
M12-CD	TTTCCTGTGCGGTCATGGTTTTTATAACCTCCTTAGAGCTCG
M13-U	AATTCGAGCTCTAAGGAGGTGGGTAGGGGCATGACCATG
M13-D	TGATGGTGATGGCTGCTGCCTCATGACGCGCCTCCGTG
M13-CU	GGCAGCAGCCATCACCATC
M13-CD	ACCTCCTTAGAGCTCGAATTCCC
R-*Nco*I	CATGCCATGGGCAGCAGCCATCACCATCATCACCACATGGAGTTAATCGGCGACAGTGATG
R-BamHI	CGCGGATCCTCAGCTTTGGGCTCTTGATCCGT

^a^: Underlined letters are restriction enzyme cut sites.

**Table 3:** The identified genes of the R-M system.

Enzymes	Name	Location	Start/stop	Length (bp)	Speculated enzymes	Predicted action sites
M1	M0K93_11245	Chromosome 1	2204207 / 2205742	1536	Cytosine-5 DNA methyltransferase	Unknown
M2	M0K93_19635	Chromosome 2	972279 / 973169	891	N4/N6 DNA methyltransferase	Unknown
M3	M0K93_01310	Chromosome 1	247810 / 248427	618	N4/N6 DNA methyltransferase	Unknown
M4	M0K93_02030	Chromosome 1	375981 / 376775	795	N4/N6 DNA methyltransferase	Unknown
M5	M0K93_02035	Chromosome 1	377350 / 379017	1668	Cytosine-5 DNA methyltransferase	Unknown
M6	M0K93_02445	Chromosome 1	459465 / 462110	2646	N4-cytosine DNA methyltransferase	Unknown
M7	M0K93_08440	Chromosome 1	1622216 / 1626595	4380	N6-adenine DNA methyltransferase	Unknown
M8	M0K93_11290	Chromosome 1	2208684 / 2210045	1361	N4/N6 DNA methyltransferase	Unknown
M9	M0K93_11445	Chromosome 1	2241244 / 2242374	1131	N4/N6 DNA methyltransferase	GANTC
M10	M0K93_16175	Chromosome 2	337789 / 338406	618	N4/N6 DNA methyltransferase	Unknown
M11	M0K93_16260	Chromosome 2	348391 / 349878	1488	Cytosine-5 dna methyltransferase	C/TGGCCG/A
M12	M0K93_17125	Chromosome 2	511042 / 511719	678	N4/N6 DNA methyltransferase	Unknown
M13	M0K93_17200	Chromosome 2	519310 / 520818	1509	DNA cytosine methyltransferase	C/TGGCCG/A
R	M0K93_11240	Chromosome 1	2203817 / 2204224	408	HNH endonuclease	Unknown
