# Supplementary material for: Identification and Characterization of an R-M System in Paracoccus denitrifican DYTN-1 to Improve the Plasmid Conjugation Transfer Efficiency
Source: J Microbiol Biotechnol. 2024 Jul 26;34(9):1826–35. doi: 10.4014/jmb.2402.02041 (PMC11473606; doi:10.4014/jmb.2402.02041)
Supplement: Supplementary file 1 [file jmb-34-9-1826-supple.pdf]

J. Microbiol. Biotechnol. 2024. 34(9): 1826-1835
https://doi.org/10.4014/jmb.2402.02041

Supplementary Table

Identification and Characterization of a Restriction Modification System in *Paracoccus denitrificans* DYTN-1 to Improve Plasmid Conjugation Transfer Efficiency

Yunpeng Shi[1,2], Wenyan Cao[1,2], Zhiping Zheng[1,2], Sha Xu[1,2], Lijuan Chai[1,2], Shenghu Zhou[1,2*], and Yu Deng[1,2*]

[1] National Engineering Research Center of Cereal Fermentation and Food Biomanufacturing, Jiangnan University, 1800 Lihu Road, Wuxi, Jiangsu 214122, P. R. China
[2]Jiangsu Provincial Research Center for Bioactive Product Processing Technology, Jiangnan University, 1800 Lihu Road, Wuxi, Jiangsu 214122, P. R. China

[*]Corresponding authors:

Yu Deng, dengyu@jiangnan.edu.cn

Shenghu Zhou, zhoush@jiangnan.edu.cn

Table S1. Nucleic acid sequences of the identified R-M system genes.

Name	Sequences (5′-3′)
M1	atgccttccactttcggcattgtcgatctgttcgccggtccgggggggacttggcgaagggttcgcttccctc<br>gtcgaggacggccatgcgccgttccggatcggcatttcggtcgagaaggaggcgtcggcccatcgga<br>ccctgacgctgcgtgcctttctgcgcgagtaccgtgcacgtcatggcgtcttgccgaaagagttcatcgat<br>ttccatgccgggctggcatccgaaccagactggtcagccgtcgataccgaagcgtggcagcatgccata<br>gacgaagcccgcgcgctcgagctcggcaccgaagctgcggcgaccgccatagatggcgccatcgcg<br>acgctgaaagcaaagtacgacgacacgatcgtgatcggcggcccgccctgtcaggcctattccctggtg<br>ggacgcgttcgctccagaggcaaggtcggttacgtcccggaggaggatgcgcggcactacctctttcg<br>cgagtacatccgggtcctcgacaagcttcgcccggccgccttcgttatggaaaacgtgaagggcatgctt<br>tcgtccaccgtcgagagccgacttgtcttcgagatgctgatggaggatctgtcctcgctcggcacgggtc<br>atgcacatcactatgaacttcgtgccgtccgggtcgaggatggcaaggccagcctgcaggaggcggca<br>cagccttcggatttcatcgtgcgcgccgaggcgtttggagttccgcagcgtcgccacagggtgatcatcg<br>tcggcatccgttcggacctcgcaggacgggccgccgatgcggagatcgctgtatccgggatggcgcg<br>gaccgtccgcgatgtcatcgaaacgatgcccgccttgcgaagcggtatcagccgcgggcgcgacgac<br>gcggctgcctggcgagaagaagtcctcgacgccgcgaagctgctggccggcatctccaaggggaag<br>gagaaacgtgcgatccgtgaagcgttcctgaccgtttcggagcgcgtgaaggaaaacccgcctatcgtt<br>cgggctgcttcacggttgccagacggctacggcacttcgaacgatgagttgctgcagtggatcgagcga<br>ccggaacttcgcgcgatcgcccagcacgagacgcgcgggcacatggcatcggatctgggtcgctacct<br>gtttgctgccgtgttcgggaccgtccgtggctacagcccgaaggccgccgattttcctctggtgctcagcc<br>ccgatcaccgcaactggcacagcggtgtgttcaatgaccgcttccgggttcagctggcggacgaggcat<br>cgaccacggtcacgagccacatctcgaaggatggccactacttcattcaccccgatcccatccagtgcc<br>ggagcctgacggttcgcgaggccgctcgtctgcagacctttcccgacgattacctgtttcttggcaaccg<br>cacgcagcagtatgtccaggtcggaaacgccgtgccgccgtttcttgcgaggcagatttcgagattgctc<br>ctttcagctttgggctcttga

atgtccccgaaacaagccttggctcttctccgctctgaccacgacgatggtgccacggttcatcgcttgga
gcgagacgcgaaagccgagatcgcttgcgagcacaatctcaagttcatgcgtcgtttgcctgacgaatcc
atgagcctgatcgtgacgtcaccgccctacaacattggaaagtcttatgaaaagcgcacatcgcaagaaa
aatatgttgaggatcaggcggcgtgcattgccgaagccgtacgtctgctcgcgccaaacgggtccatct
gctggcaggttggcaaccacgttgactcaggcgagatattcccgctagacatccttctctacccgctcttta
agcaccacggcctgcaactccgcaatcgaattgtttggacgttcggtcatgggctgcactgccagaagc
M2 gcttttcgggtaggcatgaaacgattctatggttcaccaaatctgaggactacacgttcaacctagatcctg
tccgcgtcccctcgaagtatccgaacaaaaagcacttcaaaggtccgaacaagggcgagctttccagca
atcctcttggaaagaacccttccgatgtttgggatattcccaatgtcaaatccaaccacgttgaaaagaccg
atcatccttgccagttccccatcggactggtggagagattggtgttggctttgacaaacgaaggcgagag
cgtcctcgatccttatcttggcgttggttcgtccgcgattgcagcgttgaagcatggaagacatgcctacg
gctgcgatctcgataaagcctatgtcgatattgcatgggatcgtattcaccagctgcgcgcaggtacgctc
cgcacccgcccgatgaacaaacccgtctatgaccccaatgcttag
atgcaggccttcggcaccggctcggtggatttcatcctgaccgatccgccctatgtgacccggttccgcg
accggcagggccgtaccgtcgccaatgacgacaatgcccgctggctgcgcccggccttcgcccagat
gcaccgggtcctgaaggacggcggtttctgcgtcagtttctacggctggaacaaggtggatttgttcgtg
gaggcctggaaagcggctggcttccgcatcgtcgggcatctggtgttccgcaagcgttatgcctcctcgg
cgcggttcctgcgctacgagcacgaacaggcctatctgctggccaagggcgatccggaattacctgcgc
M3 agcccgtgcccgatgtgctggatttcccctataccggcaacaggttgcacccgacgcagaagccggtgg
cggcgctgcgccggctgatcggggcgttcacccagcccggcgatctggttctggacccgttcagcggc
agcggttccacgctggcggcggcgcatctgctgggccgcgactggctcggggtggagctggacgtga
gccattaccagaccatcggcaggcggatggcggccctgcaagagcgggaccggaaggcggcggcat
ag
atgaccgtgcatcgccccatcctgcgctggcacggcggcaaatggcggctcgcgccatggatcatcga
M4 gcagatgccgcatcaccgaatctatgtcgaaccgtttggaggcgcggcctccgtgcttctgcgtaagcct

cggtcttatgccgaagtctacaatgacttggatgatgaagtcgtaaacctgtttcaggtgctgcggtcggat
cggtcgcacgacctgattgacctgctgcgactgacgcctttcgcggagagcgagttccgcactgcgtat
gaacctgcatatgatccggtcgagcgcgcccgacgcatcattatccgctctttcatgggattcggatcgaa
cggtcaccagcgcaggacgggcttccggtcgaacagcaaccgctcgggcacgacaccggcgcgcga
ctggcggaattaccccgacgcgctggcaatgatcgttgatcgcctgtcgggcgtagtgatccagaaccg
cgatgctcgtgaggtcatggcagcacacgattccccagagaccctgcattatgtcgatccgccctatgtca
tgggaacgcgggactatggttcggactatgcgcatgagatgtcggacgcggaccacatcgacctgctg
gcggcgctgggcgcgctgcgcggccgggtgatcctctctggttacgccaactccctttacgatgaagcc
ttggccggctggcagcgcttggaacgccgcgcgcatgccgatggggctcgggaaaggaccgaggtg
ctgtggatgaatttcgagccagatcggctgctttga

atggtgaacctcagctttctctcggtctgcagcggtatcgaagcggcttccctcgcatgggagccgctcg
gatggaaagccatcggctattcggagatcgagccctttccctgccacgtcctgcaccaccgcttcggtgc
cggccgtcccatcttcatgcccgcgccggacgaggcggggctttcggcaaaggaccgcaaggcccgc
gccgccgcaatccgtgccgtcgccaaactccccgaggtcggccgcgttccgaacttcggggacatgac
gcaattcgacaggtggccggatgcagctttcgatgttcttgtcggcggaaccccctgccaggactattcc
gtcgcgggacttcgcctcggcatggctgggagccgagggcaactcaccctcacatatgttgaaattgct
gcacgatatcgcccccgctggttcgtatgggagaatgtccccggcgttctcagcagcaacggcggacg
M5 ggactttgcgcgatttctgggtgaaatcagcggccagcagatcgacgtccccgatgggggctggaaga
acgccggaatcgtctccggcatccccgaagcctatggcctcgcctaccgggtgcttgacgctcagttcgt
gcgaacacgcggacaccctcgggcggtgccccagcgacggcgacgtgtgttcgttgtcggatatcttg
gagactggcgacgtgccgcagcagtattatttgaccgcgaaagcctgctcgggaatcctccgccgcgcc
ggcaattggggcaaggacttgcctctagccttacggcaagcactggaggctgtagcggcaaggacgga
atcgacggccggctgatcgctatggcccacggccaaggcggtgccgagatcggcatcgatcatggac
caaccctgacatgcaaccacgaagcccccatcgtcgctcactccctgcgcgccgagggcttcgacgcg
tccgaggacggcaccgggcgcgggacgcctatcgtgccggtcccgcagcccttgtccttccagccgg

gtaacctcgcccgtggcgcgggggccgatccaagcgcggaggttttccccactttgaaggcggatcat
ggtcgcgggctctcggatcagttcccccatgttgcccaccccattgcctttgattgcctcgctggcggcga
gacgggcctgtccatcggagacgtgcctggcgcgctccatggcggcggcaagagcgggggccggg
ccgcgatatgcttctcggccaaggattacggcgcggacgcgcaggacgatgtgacgcccacgctccgc
gccatgggccatgccgattcccatgcgaatgcgggcgggcagttggcggtcgccattcaggagcgcg
ccgtctgcgagaacccgaacgcggggcctgacggcaagggcttccgagaggacggtgcctcctacac
gctggaggcccgcaccacgccgcaggccgtcgccctgccatgggccgtgcggcgacttgtcccggaa
gagtgcgagcgcctgcagggcatgcccgacgaacacaccatgatcccgtggcggggccggaacggc
gcacctgacgggccgcgctatcgatcccttgggaattcgttcgcagtaaacgctgttgaatggattggcg
aacggatcagtatggtggagatgctgaaatga
atgggccagcatccttcctatgacgaattcctgcgcgccaaggccgccatcgcgccgcgcttcggcggc
atgcaggtggacgaggcgaccctgaaccccgccctgaaaccgcacacccgccgcatggtgagctggg
gcctgcaaggcggccggcgcgcatggttcgccagcttcggcctgcacaagaccgcgacgcagctgga
gaccatgcgcgtgctgaccgagacgacgggccagccgacgctgatcactgcgccgctgggcgttcgg
cgcgagttcatgcgcgaggcgcaggagcggttccggggctcctttgccgtggacctgaaattcatccgc
gacagcgatcaggtcgacggcccgggcatctacctgaccaattacgaatcggtgcgcgatggcaaggt
ccagcccgatccgttcatcggcgtcagcctggacgaggcggcaatcctgcgcggcttcggtggcacca
M6 agaccttccgggagttcatggcgctattcgccggcgacgaccggcgcgaccagtccaaccggatcaag
accgccggcgtcccctatcgcttcgtcgccaccgccacgcccagcccgaacgattacatcgagctgctg
tcctacgccggcttcctcgacgtgatggatatcggccaggccaagacccggtttttcaagcgcaacagcg
agaaggccgaccagctgaccctgatgcagcacaaggaacgggaattctggctgtgggtcgcgtcctgg
gcgctgttcgtcactaagcccagtgatctggacccgtcgttttcagacgagggctacgacctgccgcccc
tcgacgtgcgctggcacgaactgccggtcgatcacctcgccggcgccgcggtcgagaagagcggcca
gaaacgcatgttcaaggacgtcgcgcgcgatctgaaggtggcagcacgcgaaaaacgcaccagcatt
ggcggtcggatcgagaagctgatggagatccgcgccgaggacccggccgcgcaccggatcatctgg

cacgatctggaggccgagcgcgccgcgctggaaaagacggtgcccggcatcgccacggtttacggtt
cccaggacctcgaccagcgcgaggagctgatcggcgatttcgccgatggccgggtgcaggagctggg
cgccaagccggtgatgttgggcagcgggaccaacctgcaacggcactgcgcctggcacgtctttctcg
gcatcggtcacaagttcaatgatttcatccaggcgatccaccgcctgctgcggttcggccagcccgccga
cagcgtgcggctcgacctgatctacaccgaggctgaacgcgaaatccgccgctcgctggagcggaagt
ggcgtcagcacaacgagatggttcagaaaatgatcgacatcatcaaagagttcggcctgtccgaggtcg
cgatgcagcaggcgctggtgcgcgccttgggcgtcgagcgggtcgaggcctcggggccggattaccg
ctgcgtccacaacgattgcgtggccgagacccgcagcatgcccgatgcctcggtgcagctgatcgtcac
ctcgatcccgttcagcacccagtatgagtattcgccaaactacgcggatttcggccacaccgacgacgat
ccgcatttctggcagcagatggggttcctgatccccgagctgctgcgcgtgctggagccgggccggatc
tgcgcgatccacgtcaaggaccggatcatccccggtggcatcaacggcttcggcttccagaccctatcg
accctgcacatggattgcgtgcgcgagttccagcgccacggctgggcctatctcggtatgaagaccatc
acgaccgatgtcgtgcgcgagaacaaccagacctatcgcctgggctggtcggaacagtgcaaggacg
gcagccgcatgggctgcggcgtgccggaatacctgctgatcttccgccggccgcccagtgacaccagc
aacggctacgccgaccggccggtgaagaaggccaagaaggaatgggaccccgaggccaaggactg
gcgcaacgagggcggctatagccgcgcccgctggcagatcgatgcccacggctacatgcggtcgaat
ggcgaccggaccctgctgcccgaggaactggaggggctggacgccgaccaggtctacaaggtctgg
aaagcctacaacctccagcaggtttacgatttcgagcatcacgtccgaatcggcgaagcgctggagaag
aagggccggctgccgccgaccttcatgctcctgccaccgcatagcgcgcatcccgacgtgtggaccga
tgtcgcgcgcatgatgaccatcaacgccgagcaggcgcgcaagggcaacgagatgcacctgtgcccg
ctgcaatacgacatcgtcgaccgggccatcgcccagttcaccgagcgcggagaatgggtctacgaccc
gttcggcgggctcatgaccgtgccgttccgcgccgtgaaactcggccgcaagggcatcggggtcgaac
tgaacaagggctattggctcgacggctgcaaatacgtcgaggccgcctcgcgcgaggccggcatgcc
cagcctcttcgacctcctcgacgccgatcaacccgaaaaccagaaactgcggaggaccgcgtga

M7 atggcccgcaaaccaattaccgatatgtccgcgtggccgtcgctgagcctcgaaggcaacctgatcgcg

cctgcaatgatcgcaaagatcgatcagcgccaggcacccgagcaatcccccgaggaatacggggttcg
caaaggcctacagatccgcgaggaaatcgccaccgccttccgcgtaggccaatcgcatttcgatgccttt
gcgaagatcgaacacccctcggccgccgccacctcgcgtttcatcgccgacttcttcaaggaaaccttcg
ggtacacagacctcacggtggcagcggcaccgatcgccctgatcgcgtccgatcgtgtgcctgtcgtcg
tcgtcccgccgtccgagctacttgaccgccgcagcccgacgctctcgacggacaggtcgcgctctccg
gcctttgcccttcaggaccacctgaatgatcgggacgaggcccttggggcatagtcaccaatgggatgc
agttgcgcctcatgcgcgacaatgcctcgctgacccgtccggcctatgtcgaggccgatctggcgcaga
tgtttaccaacgaagacattgcgtccttcgcggtgctctggctcatgatccaccgcacccgcttcggcgcg
gcagagaccaccgccaccgattgccccatcgaacgctggcgcgatgccggctccaaggagggcgaa
gccgcgcgagatcgcctggccggtcaggtccaactggccctcaaactgctcggctcgggcttcctcga
ggctaaccccgacctcgccgcgaaactgcggtccggcgaagtgaacctgaccgagtggttcaacgaac
tcctgcgtctcgtctaccggctgatcttcctgatggtggccgaggaccggaacctcctccaccccgaaaa
ggccaagcccgaggcgcgcgccctttacgcccaaggctattcgctccagtcgctgcgcaagcaatgct
accgcgccgccacctgggacaagcaccatgaccgctacgaggggggtgaagatcgtcttccgcgccctc
acacatggccaacccgcccttgcgctgcccgcgctcggcggcctctttgccgaggacaggctgcccca
cttggagaccgcccgcctgcgcaaccgcgccttcatggaagcgctctatcgcctctcctggctcgcgga
caagaccggcatggtccccgtcaactggcgcgcgatggagaccgaggaactgggctcggtctacgaa
tccctcctcgaactccagccgcagctgtgtgacgacggcaagacgctggtcttcgcctccgaggcggcc
gagcagaagggcaaccagcgcaagaccaccggctcctactacacgcccgacagtctcgtgcaggcgc
tgctcgacaccgcgctcgatcccgtgctcgacaagactgaggccgaggcggacgatcccgccaaggc
gctgctgaagctttcggtcatcgaccccgcctgcggctcgggtcacttcctgctggccgctgcccgccgc
atcgcaacgcggcttgcccgcattcgtgagggcggcactccgggactcgaacacttccgccatgcgctg
cgcgatgtcgcgcgctgctgcatccacggggtggaccgcaaccctatggcggtggagctgaccaaagt
cgcgctgtggatcgagacggtggacccgggccttcccctcggcttcttcgacgcgcagatccgctgcg
gcgatgcgctgctcggcgtgttcgacctgcaggtgttgcaggacggcattcccgatgccgcctacaagc

cgctcaccggcgatgatcgaaacacggcgcgctactaccttcaagcgaaccgcgccgcgacgtctggg

caaggcgggttcgacttcggcaccggtcaggcgtcgatgcccgcgatgaagccgctggcactggattt

ctcgggtttccgcgacctgcccgaggatacggtcgagcagatcggcgccaaggccaagcggttcaag

gagctgcgcaaggggcagaccttcgtccgcgccaaggcggcggcggacctctatgtggcggccttcct

gctgccaaaagtgggcggcgcaccggcgggagcgtcggagcggacagtaccgacgaccgaagaga

tgtggatggccctcaatcagggcaagatgcgccaggcgatggtcgaggcccccaaggccgcccgcaa

cgcccgcgccctgcattggccgctggagttccctgatgtgatgcaacgcggcggcttcgacgtggtgct

gggaaatccgccttgggaagtcattcagctatcagagaaggagttctttgcgtccgtggcaccggaagtt

gccaccttggccggagcaaaacggaaggccaaaatcgcaaagcttgaagagatcaatcccgccctctt

catgtcgtatgttcttgggaagcgcgaatttgatgccgccaatgaatttgctcgcgcatcaaatcgcttttcc

tattctgccgaaggcaagctaaatacatactctctgtttgccgagctctttctttccctaatggggagcggtg

gaggcgctggcctgatcgtgccgacaggtatcgcgaccgataagttcaatgcaaagttcttcgctcatatc

agcggagaaaagatggtgcgctcgctgttttcgttcgagaatgaagagttcgtctttcccgcgattcacca

ctcaatacggttttgcctactgacgctatcccgcgctaatattgaccgggccgagtttgcatttttcctaaga

aggatatcgcagctcgaagatccaacgcgagtttaccatctctcgcccgaggaaattcagctcatcaacc

cgataacttcaacagctccaattttccgaacgagagacgatgcggacctcaccgctgcagtgtataatcg

attgccagttctcggggaggcgcttacttgggagacttggcgggttgacctaattcaaaatttcttctcgact

tcgaacgcagcagactcggaaattatagttcgcgccgaggacatgggggcaacaaccggatttgttccg

gtgctcaggggaacaatggttgaccagttcaatcacggcgccgctgcgtatgacctcacagccgacgaa

ttcatcgaaacgacgccgcttgatctgcctgagagctgggcggacgttcggtcagataagtacgttccca

ttgacgaactcgagaagaaacttcgcgccctcgattgggaagatggatggttggctggatggcgcgatat

cactagtgcccacgttaaccgtacggtgatagcgagcattttccccaaaacggcaacagacgacacccctt

tctttaatgctcccgagaggtggagctacacacgcagccacactagtagcgaacttgaattctttgcctctc

gactatgttgcgcgacaaaaggtcggcggcacgcatattcgtaagtatgtgatttcacagtttcccatcctg

ccgccagaattctactctgaattgcgcctagatttcgtgacgtctcgcgtactagaactcacctacacttcac

acaatctggcctctttcgcccgcgatctgggccatgacggcccgcccttcgcctggaacgaggaccgcc
gcgcgcaactccgcgccgatctcgacgccttctacgcccgcgcctacggcctgacccgcgacgagctg
cgctacatcctcgaccccgccgacgtgaagggccccgactacccctcggaaaccttccgcgtcctgaa
ggaaaaggaaatccgccaacacggcgaataccgcactcgccgcctcgtcctcgaagcctgggaccgg
atggcgcaggatggcacgtttgccgctctcggtatggctgacagagcaatggccgtcgctcaacaaatc
gcgctgtccccgctcaaccagtatgaggacgcaatgtgggcgtggcctcagggtatcaatgagcgaga
cagagttcgggcacagcttcgggcgatggtgggtattctgccagggccttccgatgcccgtcgcgtccg
tctggctgcactcgcctgccttcagccaggtctgctggatgggttcctgatggaagcggatcgccgagag
tggcaacgtctcttggcgcactcgacgggcgctacccaactcgttccgccaacgaacgcggcttggggc
gcggccttcaatgaacttgtttcctctgcggtgttggatgtctcatcagacggcacacaatggagcgcagg
ttcgaggcttgatcgatcgaccctgaatgcaagcgatcctgccgtcggccgcgcaatcttcgcatggtcg
aggctggaaggtgcggacttggatcacgggctctcgcaggtgtcggccgtaatccttccgttcatcagg
gagggtcgccttgcagcctga

atgactgatcgccagctgaccgtcgaataccgcggcctcgactgccttgtgccttatgcccgcaacgcgc
gcacgcattcggacgcacaggtcgccgagatcgccggctcgatccgcgagttcggcttcgtgaacccg
gtgctgattgccgaggacggaacactgatcgctggccatggccgggtgctggcggcccggcttctggg
cctgccgacggtgcccgcgatcaccttgaccggcctcagcgacagccagcgccgggcgctggtgctg
gccgacaaccgcatcgcgttgaatgcgggatgggacgaaagcctgctggcgctggaactcggcgatct
gaaggaggcgggtttcgatctcggcatcatgggcttcgaggatggggaactggaccggctgctggctg
M8
gcgccgtcgatgaggacagttcgtccccgccgccggtcaccattcccgaactgccgcgcaaccctgcct
cgcggacgggtgatctctggattctcggcgagcaccggctgctctgcggggacagcacatcccacgac
gatgtccgccggctgatgaatggcgagcgggccgtgctgtttgccaccgatccaccctatctggtggatt
acgacggctcgaaccatccgacccgcaacaaggactggtcgacctcctacggcaccacctgggacga
cagctcacagggggccgaactctacgacggcttcatcgccgccgcgttggccgaggcgatcaccgag
aatgccgcctggtattgctggcacgcctcgcgccgtcaggcgatgctggaagcctgctgggaaaaggc

cggcgccttcgtccaccagcagatcatctgggtgaaggaccggggcgttctgacccggtcgcattacct

ctggaagcacgaaccctgcctcatgggctggcggcgtccgaaccgcccgccgaaagtggccgatcag

acgctgccctcgacctgggagatgccgagtttcgccagggacgagcgccccgaccatcccacgccga

agccgctcgacgccttcggcattccgatgcgccagcacgtcgcgcgcggcgggctctgctatgagccc

ttctgcggctccgggtcgcagatcatggccggcgaggtcaacggtcggcgcgtcttcgcgatggagatc

agcccggcctatgtcgatgtcgcagtggaacgctggcaggccgagaccggcaaggacgcgatcctcg

acggcgacggtcggaccttcgccgaggtgaaggccgagcggcttggtgaacacgcaggcacggatg

cggaggcggatggcgacgctgacggtgatgcctccgcccgcaagcggcgcagccggagcaaggcg

gcatga

atgacgaagcttgacatgaaatcccgcgctgcggcgcgaacccttccgctcaaccagattctggccgga

gattgcatcgagatcatgaatgcgctgcccgaggccagcgtcgacctgatctttgccgacccgccctata

acctgcaactcaagggcgagctgcatcgaccggacaattccagggtcgatgcggtggacgaccattgg

gaccagttctcgggctttgccgcctatgaccgctttacccgcgactggcttgccgccgcgcgccgcatcc

tcaagccggacggggcgatctgggtcatcggctcgtatcacaacatcttccgggtcggcgccgagttgc

agaaccagggcttctggatcctgaacgacgtggtctggcgcaaggccaacccgatgccgaatttccgcg

gcaagcggctgaccaacgcgcatgagacgatgatctgggccagcaagtccgagggtgcgaaatacac

cttcaactacgaggcgctgaaatcgctgaacgagggcgtgcagatgcgctcggactgggtgctgccga

M9

tctgcaccggcggcgagcggctgaaggatgcgggtggcgccaaggcgcatccgacccagaagcccg

aggcgctgctgcaccgcatcctggtcggctcgaccaatccgggcgacgtggtgctggacccgttcttcg

gcaccggcaccaccggcgcggtggccaagatgctgggccgcgacttcatcggcatcgagcgcgaag

cggcctatcgcgaggtggccgagaaacgcatcgcccgcatccgcaagttcgacagcgaggccatcgc

caccatcaagcccaagcgcgccgagccgcgcgtgcccttcggccaggtggtcgagcgcggcatgctg

cgcccgggcgaagaactttactcgctgaacaaccgccacaaggccaaggtgcgcgccgatggcagcc

tgatcggcaacgacgtgaagggctcgatccatcaggtcggcgccgcgctggagggggcgccaagctg

caacggctggacctattggcacttccggcgcgaggggcggatggtgcccatcgacatcctgcgccagc

agatccgcgccgagatggaggccgacgagggccgcgcccactga

atgcaggctttcggcagcggttcggtggacttcatcctgaccgatccgccctatgtcacccgcttccgcga

ccggcagggccgcaccgtcgccaacgacgacaacgcccgctggctgcggcccgccttcgcccagat

gcaccgggtcctgaaggacggcggcttctgcgtcagtttctacggctggaacaaggtcgatttgtttgtcg

aggcgtggaaggcggccggcttccgcatcgtcgggcatctggtgttccgcaagcgttacgcctcatccg

cccgcttcctgcgctacgagcacgaacaggcctatctgctggccaagggcgacccggaatcgcccgcg

M10

cgtccggtgcccgatgtgctggactttccctataccggcaacaagctgcacccgacgcagaagccggtg

gcggcgctgcgcaggctgatcggggcgttcacgaagcccggcgatctggtgctggacccgttcagcg

gcagcggttcgaccttggcggcggcgcatctgctgggccgcgactggctcggggttgagctggatgtt

gagcattaccagaccgccggcaagcggatggcggccctgcaagagcgggaccggaaggcggcggc

ataa

atgagcgcccgtcgcacccacctttccctgccgttgcgcgtactcgacctcttcagcggcgccgcgggc

ggctggtcgctcggcctgcatcgggcgggctttgtcaccgtcgccgcctgcgagatcgtcgcatggcg

gcgcatcctctattcggagaattttccgcatgtcagactccacgaggacgtccgaaccctcacggcagct

cgacttgtttccgacctcggtggcctgccggagatcatcgtcggctcgccgccatgccaggacatctcga

gcgccaacaccaagggcaaggggatcgagggcgaacgctcgggcctctacctcgaggccgtgcggc

tggttggagagtgccgccctcgctggttcgcttttgagaacagctctgtgctcagaactcgcggcgccga

ccggctgctcgatgagctggaagcgctcggctacgcctgctggccatgcgtggtgggtgctgcggatat

M11

cggcgccaaccatgtccgcaagcggtcctggctcatcggctgcgatcccgaacagcttgccgacaccc

gtgtcgcaatcccagcagggtggattgcggctggagggcggatcgggggcgcggaaggcgatgaag

gactcgggtctttccgatctgttccgcaaccgacccttgccgacgccgatggcctcggacgggatgaag

gacggcgccggcggcggcgcgggatcgacatatccgctgcggatgatcctggcgacgccgcggaag

tccgacgcgcatcgcggtggccggggcgatctgctgagccagttgcagggccacaacagccaccatg

cgggcatgatgggcacgccgacggcgaatgcggcaccgcggggcagcgtgttgcgcaagcacaag

ggccggatgacctcggatcaggaccgtttcgaccgcagcccgacgatcggcgaagcgctgcatttcga

caggcgggtgccggaaatcctgccgacgccgacgaagcgggacagccggatggacggctggagcc
cggcctacgaccgtcggaagtcgccgaccatggatgcggtgatggacggggcgatgacggatcggg
cgccggacaaatgggccggggcgcgggcgctggcggcgatcttgcggagccatggcctgactggaa
cggcggcattgcccatcacatacgggtggatgatgggatttccgcctggctggctcgcacgcaccttgc
gctcggcagccgcaaagggacgtctgcggcgagcctgattgtcgaagccttcggcgatgcggtggtgc
cgcagatccccgaggccatcggccgcgccattctgcgcagcgaggccgcgctgaacgcggtgctcgg
tggtgctccggcgaacgatgccgctaacgacgacggtcaatccatcgacgctggccgagggaatgcc
gcgcccaacgacaatgctgtcgatgaagctgccagtggcagcccgacggaccacgggggtgcgccat
ga

M12 atgaccgcacaggaaaatacccccgcaacctcgtcatcaacggcgattgcatcgaggtgatgcaggc
gttcggcaccggatcggtggatttcatcctgaccgatccgccctatgtgacccgcttccgcgaccggcag
ggccgcaccgtggccaatgacgacaacgcccgctggctgcgcccggccttcgcccagatgcaccggg
tcctgaagcagggcggtttctgcgtcagcttctacggctggaacaaggtggatctgttcgtggaggcctg
gaaggcggcgggcttccgcatcgtcgggcatctggtgtttcgcaagcgttacgcctcctcggcgcggtt
cctgcgctacgagcacgaacaggcctatctgctggcgaagggcgatccggttctgcccgcccgtccgg
tgcccgatgtgctggattttccctataccggcaacaagctgcatccgacgcagaagccggtggcggcgc
tgcgcaggctgatcggggcgtttacccagcccggcgatctggtgctggacccgctcagcggcagcgg
ctcgaccttggcggcggcgcatctgctgggccgcgactggcttggggtggaactggacgtggcgcatt
accagaccgccggcaagcggatggcggccctgcaacagcgagaccggaaggcagcggcatag

M13 atgaccatgccgcgtgcgtctctttccctgccgctgcatgtgctcgatctgttcagcggtgccgccggcgg
ctggtctctcggcctgcaccgggcggggttcatgaccatcgccgcctgcgagatcgtggaatggcgcc
gcatcctctattcggagaattttccgcatgtcagactctacgccgatatccgcgacctcacggcaactcga
cttgtttccgacctcggctgcctgcccgacatcgtcgtcggctcgccgccctgccaggacatcagcagcg
ccaacacgaaggggaaggggatcgagggcgaacgctcgggcctctacctcgaggccgtgcgcctgg
tcggagagtgccgccctcgctggttcgcttttgagaatagctctaacttgcgaactcgcggcgccgaccg

gttgctcgatgcgctggaagcgctcggctacgcctgctggccatgcgtggtgggtgctgcggatatcgg
cgccaaccatgtccgcaagcggtcctggctcatcggctgcgacccacggcagcttgccgacacccgtg
tcgcaatcccagcagggcggcatgcggctggagggcggatcgggggcgcggaaggcgatgaggga
gtcggggctttccgagctgttccgcaatcgcccgctgccgacgccgatggcctcggacgggatgaagg
acggggcgggtggcggggcgggctcgacatatccgctgcggatgatcctggcgacgccgcggaaga
cggacgcggatcgcggcggccggggcgatctgctgagccagcttcagcgccacaacagccgccatg
cggggatgatgggcacgccgacggcgaatgcggcgccacgggggagcgtgctgcggaagcacaag
ggcaggatgacctcggatcaggaccgtttcgaccgcagcccgacgatcggcgaggcgctgcatttcga
cagaccgctgcaccgggaacccgaaaccttaccgacgccgaccaagcgcgacagccgcatggacgg
ctggagtccggcctacgaccggcgcaaatcgccgaccatggacgcggtgatggccagagcgatggat
ggtgcgatgaccgaccgggcgccggacaaatgggcgggcgcgcgggctttggcggcgatcttgcgg
agccatggcctcactggaacggcggccttgcccatcacatacgggtggatgatggggtttccgcctggc
tggctcgcacgcgcattgcgctcggcaatcgcaaggggacatctgcagccagcctgatcgtcgaggcc
ttcggcgatgcggtggtgccgcagatccccgaggccatcggccgcgccatcctgcgcagcgaggccg
cgctgaacgcggtgctgggtggcgcgccggcgagcgatgctgccgccgacgacagtccgaccatga
gcacagtcgcaggtgatgccgcgaccgacggcgatgcagtcggcaacgcgcccaccgacaatcctgc
ggaccacggaggcgcgtcatga

atggagttaatcggcgacagtgatgcggagatcctggctttcgatgaacccgaaatcgaagcgtcagaa
ggtcgaataatgacccgagttcatgtttcccgagaacgaaacagtacactcgtgaagaaaaagaaagcc
gtcgcgctgagacagcatggctgtcttgcctgtgaggtctgtggtttcgatttcttgcaaacctacggagag
R
aggggccgggaatttatcgaatgccaccacaccctgccaatttcatcgctcggtgaacacggcaagacg
aacctgaaggaccttgccttggtctgttccaactgccaccggatgatacatgctcgacgaccttggtggtc
aattgacgatgctaaggccgcgctgatcggcgccaacggatcaagagcccaaagctga
